# Preparing accessible and understandable clinical research participant information leaflets and consent forms: a set of guidelines from an expert consensus conference

**DOI:** 10.1186/s40900-021-00265-2

**Published:** 2021-05-18

**Authors:** Eleanor Coleman, Lydia O’Sullivan, Rachel Crowley, Moira Hanbidge, Seán Driver, Thilo Kroll, Aoife Kelly, Alistair Nichol, Orlaith McCarthy, Prasanth Sukumar, Peter Doran

**Affiliations:** 1 School of Medicine, University College Dublin, Dublin, Ireland and Health Research Board-Trials Methodology Research Network, National University of Ireland, Galway, Ireland; 2 School of Medicine and School of Nursing, Midwifery and Health Systems, University College Dublin, Dublin, Ireland and Health Research Board-Trials Methodology Research Network, National University of Ireland, Galway, Ireland; 3 Postal Address: Clinical Research Centre, Catherine McCauley Education and Research Centre, Dublin, Ireland; 4 School of Medicine, University College Dublin and Saint Vincent’s University Hospital Medical Research and Ethics Committee, Dublin, Ireland; 5 Patient advocate, and graduate of the Irish Platform for Patients’ Organisations, Science and Industry (IPPOSI) Patient Education Programme in Health Innovation, Dublin, Ireland; 6 National Adult Literacy Agency, Dublin, Ireland; 7 Centre for Interdisciplinary Research, Education and Innovation in Health Systems, School of Nursing, Midwifery and Health Systems, University College Dublin, Dublin, Ireland; 8 Clinical Research Centre, School of Medicine, University College Dublin, Dublin, Ireland; 9 School of Medicine, University College Dublin, Dublin, Ireland; Clinical Research Centre, School of Medicine, University College Dublin, Dublin, Ireland; Australian and New Zealand Intensive Care Research Centre, Monash University, Melbourne, Australia; 10 Department of Intensive Care, Alfred Hospital, Melbourne, Australia; 11 Saint Vincent’s University Hospital Research Ethics Committee, Dublin, Ireland; 12 Clinical Research Centre, School of Medicine, University College Dublin, Dublin, Ireland; 13 School of Medicine, University College Dublin, Dublin, Ireland; Clinical Research Centre, School of Medicine, University College Dublin, Dublin, Ireland; 14 Health Research Board-Trials Methodology Research Network, National University of Ireland, Galway, Ireland

**Keywords:** Clinical research, Clinical trials, Informed consent, Participant information leaflets, Patient information leaflets, Patient and public involvement

## Abstract

**Background:**

In line with Good Clinical Practice and the Declaration of Helsinki, it is the investigator’s responsibility to ensure that research participants are sufficiently informed, to enable the provision of informed consent. The Participant Information Leaflet/Informed Consent Form is key to facilitating this communication process. Although studies have indicated that clinical research Participant Information Leaflets/Informed Consent Forms are not optimal in terms of accessibility, there is little or no specific guidance available. The aim of this research was to propose and agree a set of guidelines for academic researchers and sponsors for preparing accessible and understandable Participant Information Leaflets/Informed Consent Forms.

**Methods:**

A literature review identified guidance for the preparation of patient-facing documents. Following critical appraisal, key recommendations were extracted and a set of recommendations which can be applied to clinical research Participant Information Leaflets/Informed Consent Forms were prepared. These recommendations were evaluated and amended by an Expert Consensus Conference consisting of a group of key stakeholders. The stakeholders included members of a Research Ethics Committee (both lay and expert), a patient advocate, experienced clinical researchers, a plain English editor and a Data Protection Officer. Consensus was reached regarding a final set of recommendations.

**Results:**

44 recommendations were agreed upon and grouped into five categories: Layout, Formatting, Content, Language and Confirming Readability. These recommendations aimed to maximize accessibility for lay participants, including readers with dyslexia, literacy or numeracy challenges, thereby improving the quality of the consent process.

**Conclusions:**

More empirical research is needed to further improve the informed consent process for research participants. However, these recommendations are informed by the current literature and have been ratified by expert stakeholders. It is hoped that these recommendations will help investigators and sponsors to consistently and efficiently produce more accessible clinical research Participant Information Leaflets/Informed Consent Forms.

**Supplementary Information:**

The online version contains supplementary material available at 10.1186/s40900-021-00265-2.

## Plain English summary

Researchers must make sure that research participants are given all of the relevant information about a research study or trial. This information helps research people to decide if they wish to take part. The Participant Information Leaflet/Informed Consent Form is an important source of information for potential research participants and their families and friends. However, Participant Information Leaflets/Informed Consent Forms are often not easy for lay readers to understand. They can be especially difficult for readers with dyslexia or those who have problems with reading, or understanding numbers. There are guidelines available for designing information leaflets for lay readers, for example, leaflets about medicines or different health problems. But it would be helpful for researchers if these guidelines were applied to clinical research Participant Information Leaflets/Informed Consent Forms.

We searched for and gathered guidance for designing and writing information leaflets for adult, lay readers and checked to see if the sources of the guidance were reliable. We then put together some recommendations for designing and writing information leaflets for research studies or trials. We formed an expert group, made up of both laypersons and researchers, who reviewed and discussed these recommendations. The expert group agreed on a final set of recommendations. We hope that these recommendations will help researchers to prepare Participant Information Leaflets/ Informed Consent Forms that are consistently easier to for participants to read and understand.

**Supplementary Information:**

The online version contains supplementary material available at 10.1186/s40900-021-00265-2.

## Background

Valid informed consent depends on disclosure of all pertinent information, capacity to consent and voluntariness [1]. In the context of clinical research, it is the investigator’s responsibility to ensure that participants are fully informed before they provide freely-given, informed consent [2]. The use of understandable and accessible written information is an important element of communication with laypersons. Written information in addition to verbal communication can improve comprehension [3, 4]. However, studies that have used a broad range of criteria have indicated that clinical research Participant Information Leaflets/Informed Consent Forms (PILs/ICFs), are not optimal in terms of accessibility and understandability [5–7]. This may lead to research participants having difficulty understanding the information contained in PIL/ICFs prior to providing their consent to take part. Concerningly, a recent systematic review indicated that research participants often have a poor understanding of some of the key concepts needed to ensure valid informed consent [8].

Clinical research PILs/ICFs pose some unique communication challenges, since research participants frequently struggle to understand complex concepts such as randomization, placebo and the risks associated with participating [9, 10]. PILs/ICFs are becoming longer [11, 12] and changes in the regulatory landscape such as the introduction of the European Union General Data Protection Regulation (GDPR) also present challenges, as additional information must be provided to participants [13, 14]. Good Clinical Practice guidelines state that ‘The language used in the oral and written about the trial, including the informed consent form, should be as non-technical as practical and should be understandable to the subject…’ [15]. However, while some general guidance on creating accessible patient-facing documents is available [16, 17], to the knowledge of the authors, this guidance has not been applied to clinical research PILs/ICFs and remains an unmet need.

The Expert Consensus Conference (ECC) methodology is defined as “*a forum for communication and participation of lay men and experts for societal decision making*” [18]. The ECC methodology is commonly used in the health technology setting and in the preparation of clinical guidelines, as a means of rapidly summarizing the available literature and gaining a consensus from an appropriate group of stakeholders [18, 19]. In particular, the ECC methodology is useful where strong evidence is lacking and therefore the insights of an expert group are required to provide guidance. There is limited empirical evidence regarding the optimal presentation and content for patient-facing documents such as layout, formatting, the use of images, language and readability. However, a review of the literature indicated many areas of common ground among organizations seeking to communicate clearly for the benefit of lay readers [16, 20, 21]. The aim of this ECC was to produce a set of guidelines for academic researchers and sponsors for the preparation of accessible and understandable clinical research PILs/ICFs and to gain consensus from an expert group of stakeholders regarding their appropriateness. Having PILs/ICF which are clearer in format and layout and contain easily understandable information for research participants will ensure that they can provide valid informed consent.

## Materials and methods

**Fig. 1:**
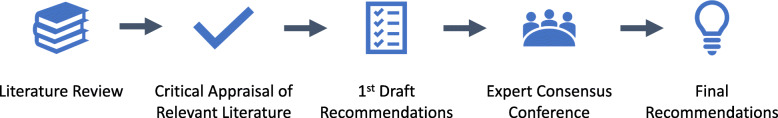
Schematic of the methodology for this Expert Consensus Conference (ECC)

The below flowchart (Fig. 1) describes the methodology followed by this ECC.

## Literature review and critical appraisal

A literature review was performed in July 2020 to identify guidance for the preparation of patient-facing documents. An initial scope of the literature indicated that guidelines relevant to this review could not be located within traditional academic databases. In fact, most of the relevant guidelines were in the form of reports from national literacy agencies, health authorities and regulatory bodies and were available on the organizations’ websites. Therefore, all entries on a Google search were examined by a single researcher (EC), using the search terms ‘Guidelines’, ‘writing’ and ‘Information Leaflets’. Following this, all reference lists were also checked. This process was independently repeated by a second researcher (LOS). The results from the search were combined and summarized by noting each result on an Excel spreadsheet. A piece of literature was deemed relevant if it purported to provide guidance on the preparation of public-facing documents for adult laypersons – this was not restricted to the medical or research context. A piece of literature was excluded if it provided guidance on preparing document for children or those with diminished or limited capacity, or wasn’t available in English. Since the majority of the guidance identified was not supported by quantitative data, each relevant document was independently appraised by two researchers (EC and LOS) using the Johanna Briggs Institute Critical Appraisal Tool for Text and Opinion [22].

### Compilation of recommendations

Guidance from each document which passed critical appraisal was extracted and tabulated. This data was synthesized and 44 recommendations were proposed from themes and findings that consistently recurred and were grouped into five categories by two researchers (EC and LOS) and reviewed by clarity and completeness by two additional researchers (RC and PD). These recommendations were circulated to the ECC participants prior to the ECC for their review.

### Expert consensus conference meeting

Eight key stakeholders were invited and agreed to take part in the ECC, which comprised of the following:

- Lay member of a Research Ethics Committee (REC)
- Expert member of a REC
- A patient advocate from the Irish Platform for Patient Organisations, Science and Industry
- A plain English editor and trainer from the National Adult Literacy Agency
- An experienced clinical research nurse
- An experienced principal investigator
- A senior academic researcher with experience in inclusive research
- An experienced qualitative researcher
- A hospital data protection officer

Efforts were made to ensure that the composition of the ECC members was balanced, including both laypersons and researchers and also persons from different operational perspectives. The lay and expert REC members and hospital data protection officer were invited from the affiliated hospital. The principal investigator, research nurse and researchers were invited from the affiliated research centre and university. The patient advocate and plain English editor/trainer were invited from their respective organisations, as listed above.

The ECC lasted for 2.5 h and took place via videoconferencing (using Zoom) in August 2020. The participants provided verbal consent to participate and for the session to be recorded. The ECC was facilitated by a Chairperson (RC), and a Discussant (LOS). The Chairperson presented each recommendation to the participants, led the discussion between the members and ensured equal representation from each participant. The Discussant presented the findings of the literature review, including the available evidence and rationale for each recommendation. During the ECC, alterations to the recommendations were proposed, and consensus was reached on each separate recommendation. For each recommendation, following discussion, a unanimous agreement was reached by the ECC participants.

### Finalizing recommendations

The amended recommendations were compiled using the ECC recording to ensure no input was omitted. The amended recommendations were circulated to the ECC participants who indicated their approval or provided further amendments. The final recommendations were then circulated and all of the ECC participants provided their approval. All approvals were documented.

### Ethical approval

This study was granted a low-risk ethical exemption by the University College Dublin Research Ethics Committee (REC) – Ref LS-E-20-94-OSullivan-Doran.

### Patient and public involvement

Two laypersons (one patient advocate and one layperson who is a member of a REC) contributed to all aspects of this ECC. Wherever applicable, this study was reported in line with the GRIPP2 Long Checklist (see supplementary file).

## Results

**Fig. 2:**
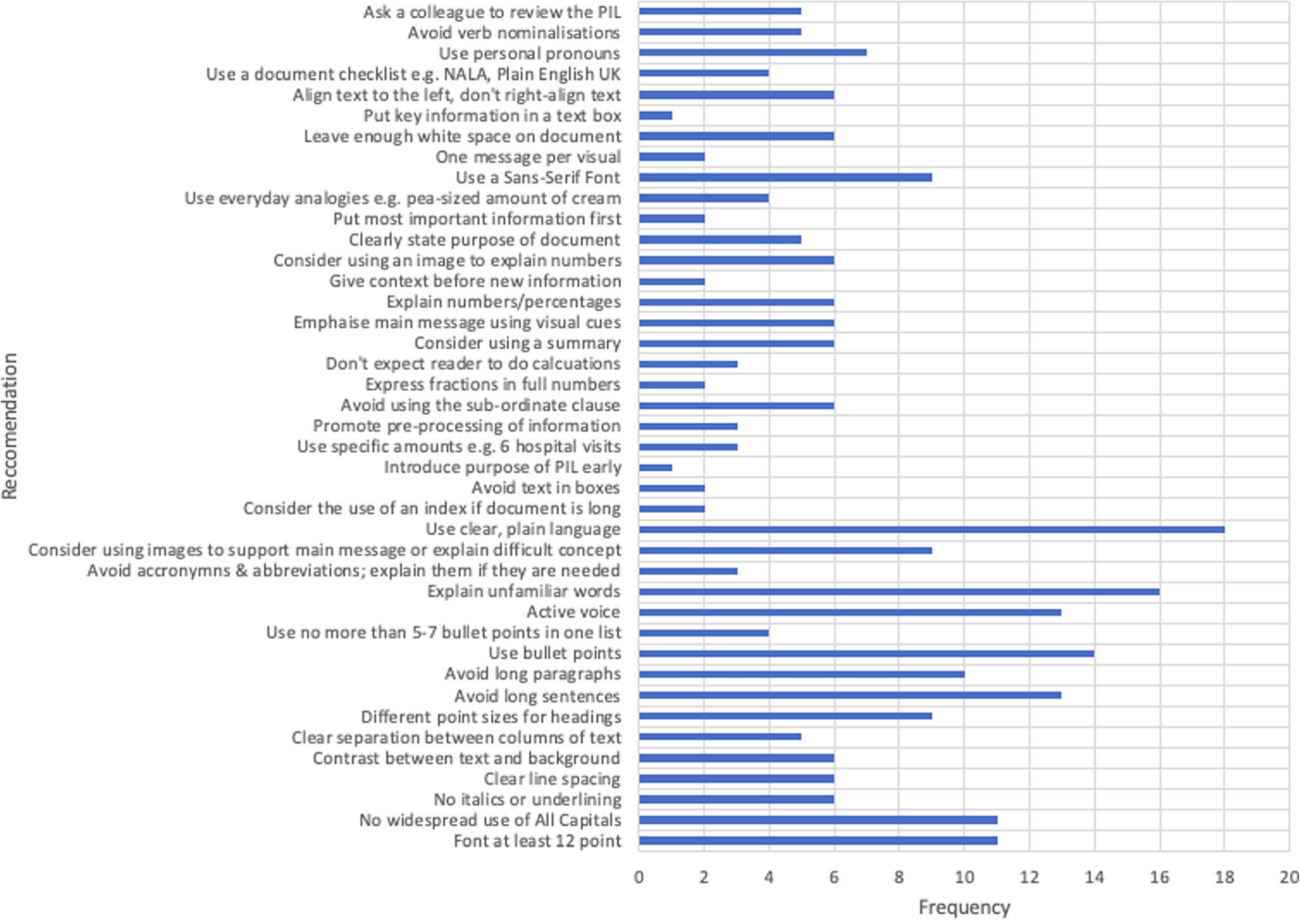
Chart of recommendations extracted from the literature search and how often each recommendation was mentioned

The searches yielded 189 (EC) and 163 (LOS) or 352 results in total. 284 documents were excluded for the following reasons: 15 (duplicates); 35 (related to paediatrics); 31 (not accessible e.g., books the researchers could not access) and 203 (not relevant - didn’t contain any useful recommendations, advertising, related to adults who lack capacity or with an intellectual disability). 68 documents were then reviewed in detail, of which 44 were excluded as they were local adaptations of national guidelines, so didn’t provide any additional insights. 24 documents underwent critical appraisal and two were excluded. Table 1 presents the 22 documents which were selected following review of the documents and critical appraisal. These documents were mainly grey literature sources – e.g., guidance produced by national literacy agencies, health authorities and regulatory bodies, along with some peer-reviewed literature. While all of the selected documents were aimed at improving clarity for adult readers in general, a number of them specifically referred to the benefits for readers with additional challenges, such as dyslexia and literacy or numeracy difficulties [16, 23–28]. Documents were published in Ireland, the United Kingdom (UK), France, the United States of America (USA) and Australia. Individual recommendations were extracted using an Excel data extraction tool. Figure 2 shows the 44 individual recommendations which were extracted and the frequency with which they were mentioned within the literature. These 44 individual recommendations were grouped into five categories.

## Final approved recommendations

In general, there was broad agreement between the ECC participants. However, there was two areas on which opinions differed. Firstly, the use of 1.2–1.5 line spacing in research PILs/ICFs can result in documents which become quite long and therefore could be off-putting for some potential research participants. Secondly, the use of readability assessment tools – it is recognised that such tools have significant limitations, however they may be of value, especially when making an initial assessment of e.g., sentence length or the percentage of passive sentences. The final recommendations, approved by the ECC, grouped into categories are as follows:

### Category 1: Layout of Participant Information Leaflet / Informed Consent Form (PIL/ICF)

#### Purpose of recommendation

The purpose of this recommendation is for the PIL/ICF to be arranged in an easy-to-read and accessible layout. Images can break up the text and help to support or explain the main message(s) of the PIL/ICF – for example to illustrate the arms of a trial after randomization. Glossy paper reflects the light, which can make the PIL/ICF harder to read. Similarly, a cluttered PIL/ICF reduces legibility. These factors are particularly important for dyslexic or neurodiverse readers, or those with literacy challenges.

**Fig. 3:**
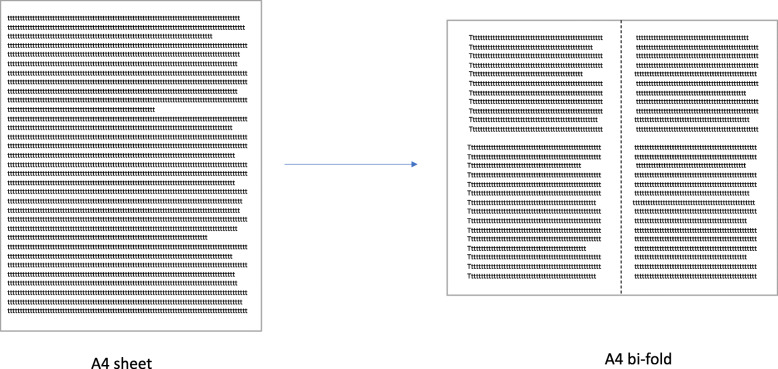
Example of A4 sheet leaflet re-formatted into an A4 bi-fold leaflet

**Fig. 4:**
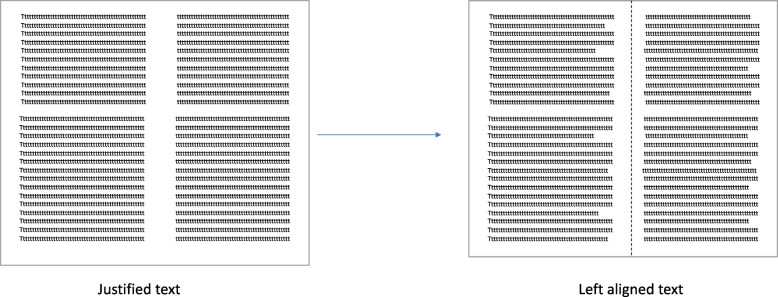
Example of justified and left-aligned text

1. **Leaflet format** – PIL/ICF should be organized into a booklet format – for example: a bi-fold booklet: see Fig. 3 below.
2. (This is a general guidance, consideration must be given to the target Participant group; if dexterity is an issue for example, an A4 page layout may be more accessible).
3. **Use of columns** – If columns are used in the PIL/ICF, there should be enough separation between them to sufficiently separate the text. This can also be achieved by a vertical line between the columns if there is a shortage of space.
4. **Line spacing** – Use sufficient spacing between lines (1.2–1.5 is recommended). A soft copy of the PIL/ICF should be available so that spacing can be altered based on the needs of the trial participant (e.g., visual impairment).
5. **Paper quality** – Use a low-to-no gloss on the leaflet’s paper.
6. **Images –** Use 1–3 simple images or illustrations when appropriate to support the main message of the PIL, or to explain a difficult concept. Each image should be clear and accompanied by a caption.
7. **Use of text boxes** – Text boxes should be used sparingly and only when appropriate to highlight important pieces of information. They should not encourage the reader to skip through the body of the text.
8. **Use left aligned text –** see Fig. 4 below.

### Category 2: Formatting of Participant Information Leaflet/Informed Consent Form (PIL/ICF)

#### Purpose of recommendation

The use of ‘All Capitals’ and underlining make it more difficult for the reader to discern the shape of the words. The text should not be too small or the reader will strain to read or avoid reading. The use of headings for each section and subsection of the leaflet is vital for breaking up blocks of text in the leaflet and making it more accessible to the reader. These factors are particularly important for dyslexic or neurodiverse readers, or those with literacy challenges.

1. **Type size** – body of text should be at least type size 12. A soft copy of the PIL/ICF should be available so that the font size can be altered based on the needs of the trial participant, for example, increased for a visually impaired participant. Similarly, if a pdf document is provided to participants, text boxes should be tested to ensure they are readable by screen readers.
2. Use a **sans serif font** (for example: Arial, Verdana, Tahoma)
3. **Use uppercase and lowercase letters** – lines of ‘All Capitals’ should be avoided.
4. **Underlining** should be avoided.
5. **Italics** should be avoided.
6. **Headings** – headings should be used and should be easily distinguished from the body of the text, using bold type face, a larger type size or a different colour. Each heading of the same level should have the same distinguishable format (for example, each section of the leaflet could be captioned by a **larger heading**, with each subsection within them having the same **smaller subheading**. The colours red or green should be avoided in the words of a heading.
7. **Contrast between text and background** – have a clear contrast in colour between the text and background and include 10–35% white space (space without text).

### Category 3: Language of Participant Information Leaflet/Informed Consent Form (PIL/ICF)

#### Purpose of recommendation

The purpose of this recommendation is to enhance the readability of the PIL/ICF and ensure that the message is as clear as possible. These factors are particularly important for dyslexic or neurodiverse readers, or those with literacy challenges.

**Fig. 5:**
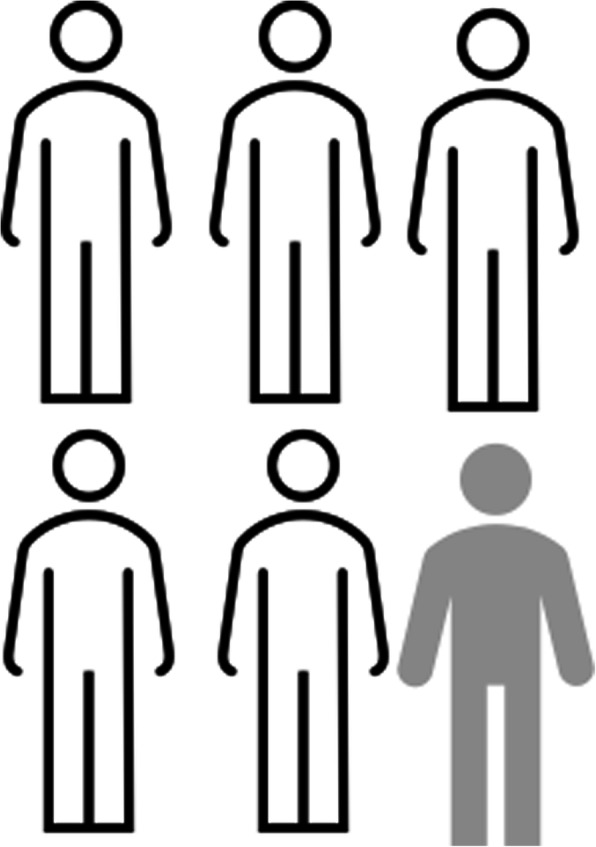
Example of stick-figure visual to explain risk

1. Minimize the use of long sentences. Aim for an average sentence length of 15–20 words. Please see Table 2 which gives an example of reducing a sentence length.
2. Don’t use long paragraphs. Break up blocks of text using subheadings.
3. Use questions in section headings for example: “*What are the risks of taking part in this study*?”
4. Use bullet points or numbered lists, rather than long sentences with lists of items, but ideally use no more than 7 bullet points.
5. Minimize the use of technical language or jargon, and where it is necessary, explain it immediately after it is used.
6. Use specific amounts, rather than words like ‘multiple’ – for example. *“As part of this study, you will need to come to the hospital six times to see the study team*”.
7. For the numbers 0–9, use their words, for 10+ use the digit, unless you are giving an example using a statistic. For example: “*1 in 6 people will get a skin rash”.*
8. Use whole numbers to explain risk or benefits – for example “*1 in 6 people will get a skin rash”,* rather than *“16.67%*”. Consider using a visual to explain risks – for example: a group of stick figures with one of them in a different colour – see Fig. 5 below.
9. Avoid adding information using a subordinate clause (or adding to a sentence with a phrase which can’t stand alone) – for example: Say “*Keep your eye drops away from sunlight. Sunlight can damage the drops*” instead of “*Keep your eye drops away from sunlight, because sunlight can damage the drops”.*

**Table 2:** Example of dividing a sentence to reduce sentence length

Long sentences	Shorter sentences
If you agree to participate, you will be requested to have an extra 5 ml of blood (1 tablespoon) taken at the 2 h timepoint of your glucose tolerance test, and for this extra blood to be analysed for some hormones which are not usually tested (insulin, c peptide and cortisol. (1 sentence, 49 words)	If you agree to take part, we will take an extra 5 ml of blood (1 tablespoon). We will take this blood at the 2-h timepoint of your glucose tolerance test. This blood will be checked for some hormones which we don’t usually test for (insulin, c-peptide and cortisol). (3 sentences; 16, 14, 18 words respectively; total of 48 words)

**Table 1:** List of key literature sources

Document Number	Document Name, Reference	Publisher or Lead Author, Year of publication or last revision
**1**	Suitability Assessment of Materials Guidance Document [29]	Doak, 1996
**2**	The A to Z of alternative words [30]	United Kingdom Plain English Campaign, 2001
**3**	Toolkit for Producing Patient Information [23]	United Kingdom Department of Health 2003
**4**	Always Read the Leaflet [24]	Medicines and Health Products Regulatory Agency, 2005
**5**	Writing about medicines for people [31]	Sless, 2006
**6**	Plain English Guidelines [16]	Irish National Adult Literacy Agency, 2008
**7**	How to produce an information brochure for patients and users of the healthcare system [32]	Haute Authorité de Santé, 2008
**8**	Guideline on the readability of the labelling and Package Leaflet of Medicinal Products [33]	European Commission, 2009
**9**	Simply Put: a guide for creating easy-to-understand materials [34]	Centers for Disease Control and Prevention, 2009
**10**	Plain Language Style Guide [25]	Health Service Executive and National Adult Literacy Agency, 2009
**11**	Health Literacy and Plain Language Overview [35]	National Partnership for Women and Families, 2009
**12**	Federal Plain Language Guidelines [36]	Government of the United States of America, 2011
**13**	Best Practice Guidance [26]	Medicines and Health Products Regulatory Agency, 2014
**14**	Patient Education Materials Assessment Tool and User’s Guide [37]	Agency for Healthcare Research and Quality, 2014
**15**	Clear Communication Index User Guide [27]	Centers for Disease Control and Prevention, 2014
**16**	Making leaflets clearer for patients [17]	The Plain English Commission, 2015
**17**	Everyday Words for Public Health Communication [38]	Centers for Disease Control and Prevention, 2016
**18**	The GET-IT glossary [39]	Moberg, 2018
**19**	How to write in Plain English [21]	Plain English United Kingdom, 2019
**20**	Guidelines for creating materials [40]	Rudd, 2019
**21**	Health literacy universal precautions toolkit [28]	Agency for Healthcare Research and Quality, 2020
**22**	Writing Plain English [20]	Government of Australia, 2020

### Category 4: Content of the of Participant Information Leaflet/Informed Consent Form (PIL/ICF)

#### Purpose of Recommendation:

The purpose of this recommendation is to enhance the readability of the PIL and ensure that the message is clear to a layperson. These factors are particularly important for dyslexic or neurodiverse readers, or those with literacy challenges.

1. Consider what information the participant would want or need to know.
2. Introduce the **purpose** of the study **early** and introduce the study from the participants’ perspective (rather than giving facts that are interesting to the researcher).
3. Use the **active voice**, wherever possible. Aim for 80–90% active verbs. For example: “*The tablets should be taken twice a day*”, rather than “*Take the tablets twice a day*”.
4. **Minimize** the use of **abbreviations or acronyms** and where they are necessary, explain them immediately after they are used.
5. Use **plain,** clear, everyday (but not sloppy) language.
6. Use **every day analogies** – for example: “*Rub a pea-sized amount of the cream into your skin”*. These must be clear and easy to interpret.
7. The main message should be emphasized with **visual cues** – larger font, headings etc.
8. **Context** should be provided before giving **new information** – for example: “*If you feel ill, phone the research team”.*
9. Consider the use of **images/graphics** to explain **numbers**.
10. Put the **image/graphic** next to the **text** that it refers to.
11. Have **one message** per **image/graphic**.

### Category 5: Checking the Readability of the of Participant Information Leaflet/Informed Consent Form (PIL/ICF)

#### Purpose of Recommendation:

The purpose of this recommendation is to ensure that PILs/ICFs are assessed prior to submission for Research Ethics Committee (REC) review. This may result in a smoother REC review and ensure that every effort has been made to prepare clear and readable PILs/ICFs for research participants.

1. **Ask a colleague** not familiar with the disease area to assess readability.
2. Carry out **user testing**, among a group with the target literacy level.
3. Use a **readability assessment tool**, such as readability software or the function in Microsoft Word to assess reading age, percentage passive voice and mean sentence length. These assessment tools are useful for guidance but have their limitations.
4. Use a **recognized checklist** – for example: the National Adult Literacy Agency Plain English checklist for documents or forms [41] Suitability Assessment of Materials [29]or Clear Communication Index [27] checklist.
5. Check that **acronyms** and **abbreviations** are **explained**.
6. Check that readers do **not** have to **perform calculations**.
  For example: Say “*Take 3 tablets every morning, afternoon and evening for a week”* instead of “*Take 21 tablets at equal intervals over a 7-day period”.*
7. Consider the use of a **summary**.
8. Check that **numbers** are **explained**.
9. Check that **images/graphics** are **explained** and **captioned**.
10. Check that the **key message** is **first**.

## Discussion

Given the paucity of clear directions around best practice in the preparation of patient facing documents, this ECC sought to identify, evaluate and apply the available literature to ensure clinical research PILs/ICFs are appropriate for enabling the consent process. The finished product is a set of clear guidelines which can be used by academic investigators and sponsors when preparing PILs/ICFs. The aim of an ECC is not to replace empirical research, but rather to provide expert opinion where there is limited evidence or indeed an evidence gap. Future research is required to evaluate whether and how well these guidelines improve participants’ understanding. Synthesizing and condensing all of the available evidence into this set of guidelines provides researchers with a time-efficient guide which can help to ensure that all PILs/ICFs are consistently more accessible and understandable, particularly for readers with literacy challenges. These guidelines could also form the basis of Standard Operating Procedures for sponsors for the preparation of PILs/ICFs, as part of a training resource for new researchers or when revising an institutional PIL/ICF template.

### Limitations

A limitation of this study is the lack of empirical evidence, e.g., randomized trials or studies within trials, regarding what factors improve readability and understandability for research participants. Therefore, the study relied on guidance produced by national literacy agencies, regulatory bodies and health agencies for patient-facing documents, and on a group of key stakeholders to adapt these to the clinical research context. Another limitation is the nature of the literature review used in this study, as any non-systematic literature review involves a higher risk of bias and a greater chance of missing relevant sources. In particular, the use of Google may lead to results which are not reproducible.

Efforts were made to ensure this group of stakeholders was balanced in terms of diversity of backgrounds. However, the group was limited to eight persons, so the size of the group is another limitation. It is also recognised that non-native readers or speakers of the local language will experience additional challenges. While many of the recommendations from this research will apply to readers in any language, e.g., formatting and layout, specific advise for this group of participants is outside of the scope of this study. Finally, this piece of work applies to PILs/ICFs which are presented in paper format. Given the movement by some researchers toward multimedia tools and E-consent, additional consideration should be given to ensure these forms of communication are clear and accessible to users.

## Conclusions

Further empirical research is required to identify what factors would improve the informed consent process for research participants. However, this list of recommendations is informed by the current literature and has been ratified by a group of expert stakeholders. It is hoped that these recommendations will help investigators and sponsors to produce more accessible and understandable clinical research PILs/ICFs in a time-efficient and consistent manner.

## Supplementary Information

**Additional file 1.** GRIPP2_Long Checklist.

## Data Availability

Data will be made available by contacting the corresponding author.

## Abbreviations

ECC: Expert Consensus Conference
GDPR: General Data Protection Regulations
PIL/ICF: Participant Information Leaflet/Informed Consent Form
